# Improvement of EMG Pattern Recognition Model Performance in Repeated Uses by Combining Feature Selection and Incremental Transfer Learning

**DOI:** 10.3389/fnbot.2021.699174

**Published:** 2021-06-14

**Authors:** Qi Li, Anyuan Zhang, Zhenlan Li, Yan Wu

**Affiliations:** ^1^School of Computer Science and Technology, Changchun University of Science and Technology, Changchun, China; ^2^Department of Physical Medicine and Rehabilitation, The First Hospital of Jilin University, Changchun, China

**Keywords:** EMG, pattern recognition, feature selection, incremental support vector machine, TrAdaBoost

## Abstract

Electromyography (EMG) pattern recognition is one of the widely used methods to control the rehabilitation robots and prostheses. However, the changes in the distribution of EMG data due to electrodes shifting results in classification decline, which hinders its clinical application in repeated uses. Adaptive learning can solve this problem but takes additional time. To address this, an efficient scheme is developed by comparing the performance of 12 combinations of three feature selection methods [no feature selection (NFS), sequential forward search (SFS), and particle swarm optimization (PSO)] and four classification methods [non-adaptive support vector machine (N-SVM), incremental SVM (I-SVM), SVM based on TrAdaBoost (T-SVM), and I-SVM based on TrAdaBoost (TI-SVM)] in the classification of EMG data of 12 subjects for 5 consecutive days. Our results showed that TI-SVM achieved the highest classification accuracy among the classification methods (*p* < 0.05). The SFS method achieved the same classification accuracy as that of the scheme trained with the feature vectors selected by the NFS method (*p* = 0.999) while achieving a lower training time than that of TI-SVM combined with the NFS method (*p* = 0.043). Although the PSO method outperformed the NFS and SFS methods by achieving reduced training and response times (*p* < 0.05), the PSO method achieved a considerably lower classification accuracy than that of the scheme trained with the feature vectors selected by the NFS (*p* = 0.001) or SFS (*p* = 0.001) method. Furthermore, TI-SVM combined with the SFS method outperformed the CNN method with fine-tuning in classification accuracy on a small data set (*p* = 0.001). The results indicate that TI-SVM combined with the SFS method is suitable for improving the performance of EMG pattern recognition in repeated uses.

## Introduction

Electromyography (EMG) pattern recognition is widely used in myoelectric control devices, such as rehabilitation robots (Lunardini et al., 2016) and prostheses (Farmer et al., 2014). This scheme enables rehabilitation robots and prostheses to perform the user's actions by extracting the movement intention implied in the multichannel EMG signals (Al-Quraishi et al., 2017; Yang et al., 2017; Teramae et al., 2018). Previous studies have attempted to identify the most suitable scheme by extracting EMG features of high separability through finding an appropriate feature selection method and an effective classification method (Phinyomark et al., 2010; Guo et al., 2015). These studies reported high classification accuracy above 90% in a laboratory environment. However, their clinical applicability is still limited in daily life (Simao et al., 2019). The reason for this limitation is that the donning and doffing of the electrodes in every use change the positions and skin impedance of the electrodes, which leads to differences in distribution of acquired EMG data. The model trained in an earlier use exhibits poor performance in a different use because of the electrodes shifting (Young et al., 2012). Re-selecting features and retraining the model of a myoelectric control device before every use can solve this problem. However, these processing methods increase the time-consuming burden on users.

Various studies have attempted to solve the problem of performance decline in models due to the differences in data distribution caused by electrodes shifting across repeated uses. Some studies attempted to extract an invariant EMG feature of specific motions to strengthen the robustness of the EMG pattern recognition model and improve the classification accuracy in repeated uses (Boostani and Moradi, 2003; Tkach et al., 2010; Phinyomark et al., 2013). Some researchers adopted an unsupervised adaptive classification method, which enables the model to adapt EMG data with different distributions (Liu, 2015; Huang et al., 2017; Prahm et al., 2019). Although extracting an invariant EMG feature or applying an unsupervised adaptive classification method can improve the classification accuracy of schemes to an extent, the classification accuracy achieved by these methods cannot satisfy the requirements under the conditions of changes of electrode positions in repeated uses. In addition, supervised adaptive classification methods have been adopted to address the problem of the decrease in the classification accuracy of an EMG pattern recognition scheme across every use (Liu et al., 2016; Vidovic et al., 2016; Ameri et al., 2020). This method was used successfully in deep learning and achieved satisfactory performance. For example, Ameri et al. (2020) proposed a convolutional neural network (CNN) with a fine-tuning method to reduce the influence of electrode shifting on the classification accuracy of an EMG pattern recognition scheme. However, a CNN model requires a huge amount of data for training in order to avoid overfitting (Phinyomark and Scheme, 2018). An incremental support vector machine (I-SVM) was proposed to improve the classification accuracy of an EMG pattern recognition scheme when the data distribution changed. I-SVM is a supervised adaptive classification method in which the classification model is updated according to data with labels from a new use, thereby improving the performance of the EMG pattern recognition scheme in repeated uses (Liang and Li, 2009; Xu et al., 2014; Liu, 2015; Huang et al., 2017). However, the distribution of data from a new use is changed because of the electrodes shifting (Gama et al., 2014). After the data distribution changes, the outdated data from an earlier use have a different distribution from the data in the new use and hinder the adaptation process of I-SVM in the new use (Huang et al., 2017). Transfer learning methods have been proposed to reduce the influence of data distribution changes on EMG pattern recognition (Pan and Yang, 2010; Matasci et al., 2012; Wei et al., 2015; Jayaram et al., 2016; Hossain et al., 2018; Li et al., 2021). For example, SVM with the TrAdaBoost algorithm (T-SVM) has reduced the data distribution differences by discarding the outdated data from an earlier use to improve pattern recognition performance compared with a typical SVM (Matasci et al., 2012). Inspired by this, we propose a novel adaptive classification method by combining TrAdaBoost and I-SVM (TI-SVM) to improve the performance of I-SVM. The performance of TI-SVM was verified by comparing it with those of non-adaptive SVM (N-SVM), I-SVM, and T-SVM.

In addition, the dimension reduction performance of two widely used feature selection methods [sequential forward search (SFS) and particle swarm optimization (PSO)], as well as that of no feature selection (NFS), was compared to find a proper feature selection method to avoid re-selecting features in different uses. The robustness of the features selected by different feature selection methods is an important element of the scheme's performance. However, previous studies rarely evaluated the performance of the features selected by the feature selection method in repeated uses (Nazarpour et al., 2007; Liu, 2014; Adewuyi et al., 2016; Zhou et al., 2016; Purushothaman and Vikas, 2018). The performance of the features selected by these methods was therefore investigated.

In this study, we developed a proper scheme to improve the performance of EMG pattern recognition in repeated uses by comparing the performance of the combinations of three feature selection methods (NFS, SFS, and PSO) and four classification methods (N-SVM, I-SVM, T-SVM, and TI-SVM) in the classification of EMG data of 5 consecutive days. The classification accuracy, training time, and response time of the 12 combinations were evaluated for data of 5 consecutive days. To evaluate the effectiveness of the scheme we developed, the classification accuracy of the scheme was also compared with that of CNN with fine-tuning, which was the most effective method previously reported for solving the problem of classification accuracy decline due to electrodes shifting.

## Materials and Methods

**Figure 1:**
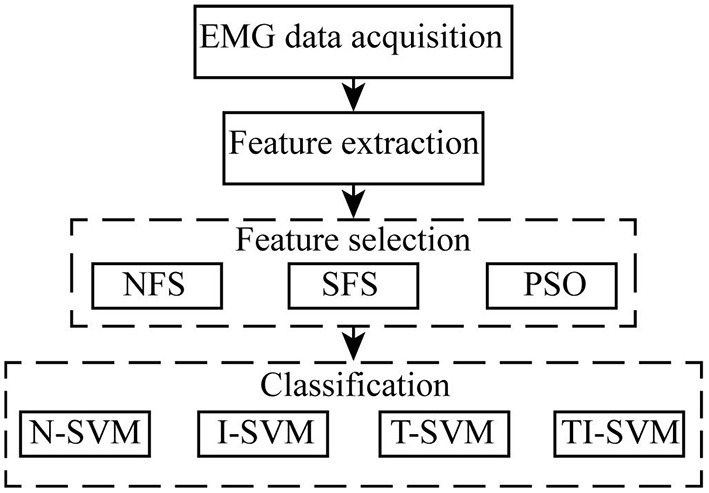
Schematic illustrating the optimization process in this study.

EMG data acquisition, feature extraction, feature selection, and classification were adopted to construct an EMG pattern recognition scheme. We mainly focused on improving the performance of the EMG pattern recognition scheme in different uses by optimizing the feature selection method and classification method. The optimization process is shown as follow (Figure 1). The performance of 12 combinations of three feature selection methods and four classification methods was compared to identify a proper scheme that provides robust performance for EMG pattern recognition in repeated uses.

### EMG Data Acquisition

EMG signals were acquired from 12 healthy subjects using a commercial system (NORAXON Desktop DTS) with seven channels. We conducted an experiment for 5 consecutive days to simulate the different uses of the EMG pattern recognition scheme. Before the experiment, all subjects were fully informed about the experimental procedure. All the subjects have been informed and signed informed consent before the experiments. The study was approved by the ethics committee of Changchun University of Science and Technology (CUST), 20190013, August 3, 2019.

**Figure 2:**
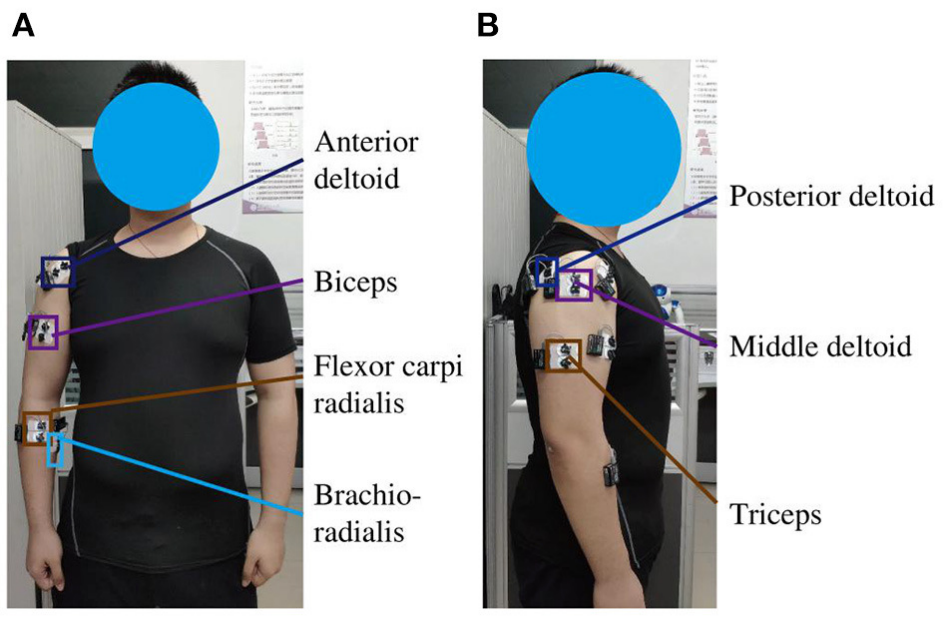
Experimental configuration of EMG sensors with dual electrodes on the right arm of subjects. **(A)** Positions of sensors on the anterior deltoid, biceps, brachioradialis, and flexor carpi radialis. **(B)** Positions of sensors on the middle deltoid, posterior deltoid, and triceps.

EMG signals were recorded from seven positions corresponding to the following muscles: the anterior deltoid, middle deltoid, posterior deltoid, biceps, triceps, brachioradialis, and flexor carpi radialis (Figure 2). Before placing the electrodes, 75% alcohol was used to clean the skin at the required locations to reduce the impedance between the skin and the electrodes. The EMG signals were sampled at 1500 Hz and band-pass filtered between 20 and 450 Hz with a 50 Hz notch filter.

**Figure 3:**
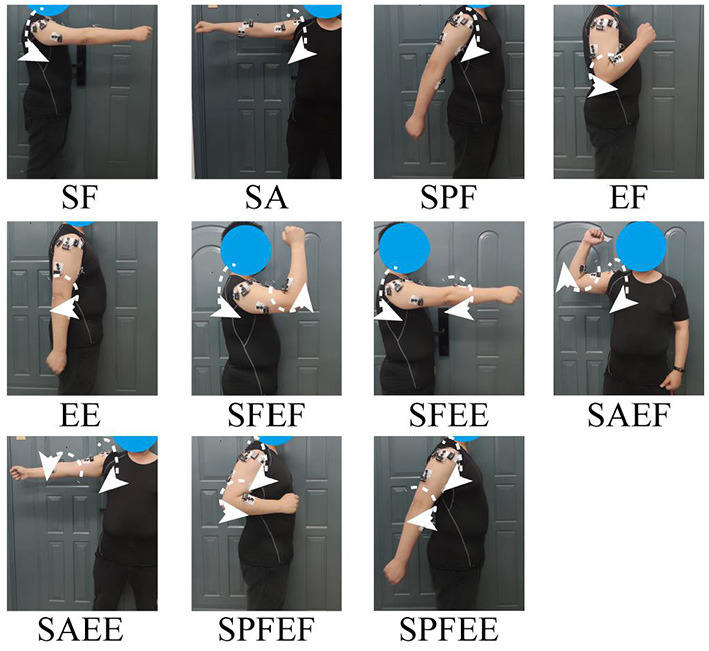
Examples of the trajectories of the 11 motions performed by the subjects in this study.

On each day of the acquisition, all subjects were asked to stand in front of a computer screen and keep their right hand relaxed. All subjects were asked to perform 11 different motions: shoulder flexion (SF), shoulder abduction (SA), shoulder posterior flexion (SPF), elbow flexion (EF), elbow extension (EE), shoulder flexion, and elbow flexion (SFEF), shoulder flexion and elbow extension (SFEE), shoulder abduction and elbow flexion (SAEF), shoulder abduction and elbow extension (SAEE), shoulder posterior flexion and elbow flexion (SPFEF), and shoulder posterior flexion and elbow extension (SPFEE) (Figure 3). The EMG data from one repetition of one motion was treated as a trial and each motion was repeated five times. Each trial lasted for 7 s and was followed by a 5-s rest period. In order to ensure the stability of the acquired signal, we selected the data of the central 5 s of each trial for subsequent analysis. Overall, 55 trials (11 motions × 5 trials) were acquired from each subject for each day.

All data were processed on a computer with Intel Core i7-8700 CPU, NVIDIA Quadro P620 GPU, and 256 GB of RAM.

### Feature Extraction

All trials were segmented using a 250-ms sliding window with a 50-ms overlap. Thus, each trial was divided into 96 overlapping segments. In total, 5,280 segments (11 motions × 5 trials × 96 windows) were obtained each day. Fourteen commonly used EMG features—nine time-domain (TD) features, two frequency-domain (FD) features, and three time-frequency (TF) features—were extracted from these sliding windows (Tkach et al., 2010; Phinyomark et al., 2013; Zhang et al., 2017; Gu et al., 2018). The nine TD features were mean absolute value (MAV), variance (VAR), root mean square (RMS), slope sign change (SSC), zero crossing (ZC), waveform length (WL), fifth-order auto-regressive model (AR5), sixth-order auto-regressive model (AR6), and cepstral coefficient (CC) (Tkach et al., 2010; Phinyomark et al., 2013). The two FD features were mean frequency (MNF) and median frequency (MDF) (Zhang et al., 2017). The three TF features were wavelet transform waveform length (WTWL), wavelet transform variance (WTVAR), and wavelet transform mean absolute value (WTMAV) (Gu et al., 2018). The 14 EMG features are detailed as next.

#### MAV

(1) $$\begin{array}{l} MAV\;=\;\frac{1}{N}\overset{N}{\underset{i\;=\;1}{\sum }}|x_{i}| \end{array}$$

Where *x*_*i*_ is the *i*^*th*^ EMG sample of a sliding window. *N* is the number of samples in a sliding window. Since the window length was 250 ms, *N* was set to 375.

#### VAR

(2) $$\begin{array}{l} VAR=\frac{1}{N-1}\overset{N}{\underset{i=1}{\sum }}x_{i}^{2} \end{array}$$

Where *x*_*i*_ represents the *i*^*th*^ EMG sample of a sliding window and *N* represents the number of EMG samples in a sliding window. *N* was set to 375.

#### RMS

(3) $$\begin{array}{l} RMS=\sqrt{\frac{1}{N}\overset{N}{\underset{i=1}{\sum }}x_{i}^{2}} \end{array}$$

Where *x*_*i*_ represents the *i*^*th*^ EMG sample of a sliding window, *N* represents the number of EMG samples in a sliding window. *N* was set to 375.

#### SSC

(4) $$\begin{array}{l} SSC=\overset{N}{\underset{i=2}{\sum }}\left[f\left[\left(x_{i}-x_{i-1}\right)\times \left(x_{i}-x_{i+1}\right)\right]\right],\\ \;f\left(x\right)=\{\begin{array}{cc} 1, & if\;x\geq threshold\\ 0, & otherwise. \end{array} \end{array}$$

Where *x*_*i*_ represents the *i*^*th*^ EMG sample of a sliding window. *N* is the number of EMG samples in a sliding window. *N* was set to 375 and the *threshold* was set to 10.

#### ZC

(5) $$\begin{array}{l} ZC=\overset{N-1}{\underset{i=1}{\sum }}\left[sgn\left(x_{i}\times x_{i+1}\right)⋂|x_{i}-x_{i+1}|\geq threshold\right],\\ \;sgn\left(x\right)=\{\begin{array}{cc} 1, & if\;x\;<\;0\\ 0, & otherwise. \end{array} \end{array}$$

Where *x*_*i*_ represents the *i*^*th*^ EMG sample of a sliding window and *N* represents the number of EMG samples in a sliding window. *N* was set to 375 and the *threshold* was set to 25.

#### WL

(6) $$\begin{array}{l} WL=\overset{N}{\underset{i=1}{\sum }}|x_{i+1}-x_{i}| \end{array}$$

Where *x*_*i*_ represents the *i*^*th*^ EMG sample of a sliding window and *N* represents the number of EMG samples in a sliding window. N was equal to 375 in this study.

#### Auto-Regression Model (AR)

(7) $$\begin{array}{l} x_{i}=\overset{p}{\underset{k=1}{\sum }}a_{k}x_{i-1}+e_{i} \end{array}$$

Where *a*_*k*_ represents AR coefficients, *p* is the order of the AR model, and *e*_*i*_ is the white noise. In this study, the coefficients of AR5 and AR6 models were extracted.

#### CC

(8) $$\begin{array}{l} c_{1}={-a}_{1},c_{k}={-a}_{k}-\overset{k-1}{\underset{i=1}{\sum }}\left(1-\frac{i}{k}\right)a_{k}c_{k-1} \end{array}$$

Where *a*_*k*_ represents AR coefficients, k represents the order of the CC coefficients. Where *c*_*k*_ represents CC coefficients. In this study, k was set to 5.

#### MNF

(9) $$\begin{array}{l} MNF=\frac{\sum_{i}^{N}f_{i}p_{i}}{\overset{N}{\underset{i}{\sum }}p_{i}} \end{array}$$

Where *f*_*i*_ is the frequency value of the spectrum at the frequency bin *i*, *p*_*i*_ is the EMG power spectrum at the *i*^*th*^ frequency bin, *N* is the number of frequency bins. Boxcar window was used to calculate the power spectrum of the signal. The length of the Boxcar window was set to 375.

#### MDF

(10) $$\begin{array}{l} MDF=\frac{1}{2}\overset{N}{\underset{i=1}{\sum }}p_{i} \end{array}$$

Where *p*_*i*_ is the EMG power spectrum at the *i*^*th*^ frequency bin, *N* is the number of frequency bins. Boxcar window was used to calculate the power spectrum of the signal. The length of Boxcar window was set to 375.

#### Wavelet Transform (WT)

WT is widely used as a tool to analyze the TF characteristics of the EMG signal. It can provide the TD and FD information of the signal at different scales. In this study, five-level wavelet decomposition was employed using the Daubechies2 (Db2) algorithm. After wavelet decomposition of the EMG signal of a sliding window, the cA5, cD1, cD2, cD3, cD4, and cD5 coefficients were selected. Finally, the WTWL, WTVAR, and WTMAV features were extracted from these coefficients.

### Feature Selection

The dimension reduction performance of three feature selection methods (NFS, SFS, and PSO) were compared to select a suitable feature selection method to shorten the training time and response time of the EMG pattern recognition scheme in repeated uses.

#### NFS

NFS served as a no-selection baseline method to evaluate the effectiveness of the other two feature selection methods.

#### SFS

In the SFS feature selection method, a Fisher's discriminant ratio *J*3 value was used to sort the separability of 14 EMG features in descending order (Nazarpour et al., 2007). To calculate the *J*3 value, three types of matrices were defined: within-class scatter matrix, between-class scatter matrix, and mixture matrix.

A within-class scatter matrix can be described using (1):

(11) $$\begin{array}{l} S_{w}=\overset{M}{\underset{i=1}{\sum }}\frac{1}{M}E\left[\left(x-u_{i}\right){\left(x-u_{i}\right)}^{T}\right] \end{array}$$

where *M* represents the total number of classes, *x* represents the feature vectors, and *u*_*i*_ represents the mean value of the feature vector of class *i*.

A between-class scatter matrix can be described using Equation (2):

(12) $$\begin{array}{l} S_{b}=\overset{M}{\underset{i=1}{\sum }}\frac{1}{M}E\left[\left(u_{i}-u_{0}\right){\left(u_{i}-u_{0}\right)}^{T}\right] \end{array}$$

where *u*_0_ represents the global mean value of all feature vectors.

A mixture scatter matrix can be described using Equation (3):

(13) $$\begin{array}{l} S_{m}=S_{b}+S_{w} \end{array}$$

Finally, the *J*3 value can be defined as follows:

(14) $$\begin{array}{l} J3=trace\left(S_{w}^{-1}S_{m}\right) \end{array}$$

The greater the *J*3 value is, the better the separability of the EMG feature is.

In the SFS method, we reconstructed the original feature vectors by selecting the most informative features from the 14 features. First, the original feature vectors were divided into a different fourteen-feature vector set according to the 14 EMG features. Then, the *J*3 value of each EMG feature vector set was calculated. Subsequently, two feature sets were established: Set A and Set B. Set A was an empty set and Set B contained the fourteen-feature vector sets sorted in descending order of *J*3 values. The SFS method selected the feature vector set in Set B that had the highest *J*3 value and moved it to Set A as the first feature vector set in Set A. Then, the SFS method iteratively paired each of the remaining feature vector sets in Set B with all the feature vector sets in Set A. The feature vector set in Set B that was paired with all the feature vector sets in Set A and produced the highest *J*3 value was identified and moved to Set A. In each iteration, one feature vector set in Set B was selected and moved to Set A as the most informative feature vector set. When each of the remaining feature vector sets in Set B was paired with those in Set A and could not increase the *J*3 value, the SFS method was stopped. The feature vector sets in Set A were then linearly combined as the final feature vector set.

#### PSO

PSO is a feature selection method based on the movement of birds in search of food (Purushothaman and Vikas, 2018). PSO creates a swarm of particles in high-dimensional space; each particle has its own position and velocity. Each particle moves to the global best position and the local best position to iteratively update its position and velocity. The update process of position and velocity of each particle is described as follows:

(15) $$\begin{array}{r} x_{j}\left(t+1\right)=x_{j}\left(t\right)+v_{j}\left(t+1\right) \end{array}$$

(16) $$\begin{array}{l} v_{j}\left(t+1\right)=wv_{j}\left(t\right)+c_{1}rand_{1}\left(p_{best}-x_{j}\left(t\right)\right)\\ +\;c_{2}rand_{2}\left(g_{best}-x_{j}\left(t\right)\right) \end{array}$$

where *t* is the number of iterations; *w* represents the constriction factor set to 0.7; *c*_1_ and *c*_2_ represent the learning factors and were both set to 2 in this study; *rand*_1_ and *rand*_2_ represent random numbers between 0 and 1; *x*_*j*_ represents the position of the *j* th particle; *v*_*j*_ represents the velocity of the *j* th particle; *p*_*best*_ represents the local best position of the *j* th particle; and *g*_*best*_ represents the global best position of the *j* th particle.

In the PSO method, the 14 features were linearly constructed as a high-dimensional feature vector. Among the 14 features, the dimension number of the feature vectors of MAV, VAR, RMS, SSC, ZC, WL, MNF, and MDF features were 1; the dimension number of the feature vectors of AR5 and CC were 5; and the dimension number of the feature vectors of AR6, WTMAV, WTWL, and WTVAR were 6. Considering the number of channels, a 294-dimensional feature vector was obtained from each sliding window. In this study, each particle searched in a 294-dimensional space to find a suitable dimension of EMG feature vectors that produces the maximum classification accuracy. The numbers of the particles and iterations were chosen from various tests to determine which achieved the best result. The number of particles was set to 80. The maximum number of iterations was 50. However, this performance may be enhanced by a dynamic PSO method.

After the feature selection, a new feature vector was obtained from the original feature vectors of Day 1. In order to evaluate the effectiveness of the new feature vectors, 5280 feature vectors from Day 1 were divided into two different data sets: four-fifths of the feature vectors served as a training set, and one-fifth of the feature vectors served as a validation set. An SVM classification model was trained with the training set. The classification model was used to classify the vectors of the validation set, and the classification accuracy was used as the criterion for evaluating the selected feature vectors for the EMG pattern recognition scheme.

### Classification

The performances of the four classification methods based on SVM were compared (i.e., N-SVM, I-SVM, T-SVM, and TI-SVM). The linear kernel function was used as the kernel function of the four classification methods. N-SVM served as a baseline method to evaluate the performance of its adapted version. Three adaptive classification methods (I-SVM, T-SVM, and TI-SVM) were adopted to adapt the N-SVM model trained using the training set of Day 1. Days 2–5 served as the target days. The EMG data from the target days were collected to simulate the application condition of the EMG pattern recognition scheme in a new use after donning and doffing of the electrodes. The dimensions of the feature vectors extracted from the data of Days 2–5 were selected according to the dimensions of the original feature vectors selected by the corresponding feature selection methods on Day 1. To test the performance of different classification methods, a five-fold cross-validation was conducted on each target day. A reverse leave-out cross-validation was conducted by using one-fifth of the data from each target day as a calibration set to adapt the model and the remaining data from the same day as a test set to evaluate the performance of the adapted model. The classification methods are described as follows.

#### N-SVM

N-SVM was adopted as the baseline method for comparison with its adapted version. N-SVM was trained by only using the training set of Day 1. There was no adaptation to N-SVM. Its performance was evaluated on the test set of each target day.

#### I-SVM

The I-SVM model was obtained by incrementally adapting the N-SVM model using the calibration set of a target day. First, an N-SVM model was trained using the training set from Day 1. Then, a typical strategy was used to adapt the N-SVM using the samples from the calibration set. The calibration set was segmented into different batches. The batch size was set to 48 by conducting a large number of experiments. Because the calibration set of each target day had 1,056 feature vectors (one-fifth of 5,280 vectors of a target day), there were 22 batches in the calibration set. The first batch of data was combined with the support vector samples of the training set of Day 1 to adapt the model. The new support vector samples were obtained from the adapted model and combined with the data of the next batch to adapt a new model again. After all batches participated in the adaptation, the performance of the adapted model was evaluated using the test sets of the same target day.

#### T-SVM

T-SVM is an adaptive classification method based on the TrAdaBoost algorithm (Matasci et al., 2012).

First, the data from the training set of Day 1 and the calibration set of a target day were weighted. Then, these data were combined into a new training set to train a new model. Subsequently, the new model was used to classify the data from the training set of Day 1. The weight of wrongly classified data of the training set was reduced, and the low-weight data in the training set was discarded while the training set was updated. The data of the calibration set were also classified by the new model. The weight of the data from the calibration set was increased if the data was wrongly classified by the model. The data with higher weight in the calibration set would be combined with the updated training set to form a new training set to train a new model. Then, the new model was used to classify the updated training set and the original calibration set to construct a new training set again. After n adaptations, n models were obtained. According to various experiments conducted, n was set to 26 in this study. From the last half of the n models, we selected the model with the most data selected from the calibration set as the adapted model. The performance of the adapted model was evaluated using the test set of the same target day.

#### TI-SVM

Both the TrAdaBoost algorithm and I-SVM were used in this model. First, the training set of Day 1 and the calibration set of a target day were used to construct a new training set using the TrAdaBoost algorithm. Then, a new SVM model was trained using the new training set. Finally, the new model was incrementally adapted using the calibration set of each corresponding target day. The performance of the adapted model was evaluated using the test set of the same target day.

### CNN With Fine-Tuning

To evaluate the effectiveness of the developed scheme, we compared its classification accuracy with that of the CNN with fine-tuning. A CNN with fine-tuning is the most satisfactory method previously reported in the literature to solve the problem of EMG pattern recognition classification accuracy decline caused by electrodes shifting (Ameri et al., 2020). The structure of the CNN is presented in Ameri et al. (2020), which was inspired by GoogLeNet (inception V3). An input picture was constructed from the raw EMG data of a 250-ms sliding window from seven channels. Four-fifths of EMG data from Day 1 served as a training set to train an initial CNN model, and the rest of the EMG data from Day 1 served as a validation set to validate the effectiveness of the CNN model. One-fifth of EMG data from a target day served as a calibration set to fine-tune the CNN model trained on Day 1. Four-fifths of the EMG data from the same target day served as a test set to evaluate the effectiveness of the fine-tuned CNN model on that target day. A five-fold cross-validation was also conducted to evaluate the performance of the CNN with fine-tuning.

### Performance Index and Statistical Analysis

There were three performance indices for evaluating the performance of the schemes: classification accuracy, training time, and response time. The classification accuracy of a scheme was the proportion of the feature vectors of the test set from each target day that were correctly classified by the scheme. The training time for the schemes using the I-SVM, T-SVM, or TI-SVM model included the time for training the initial SVM model using the training set of Day 1 and the time for adapting the initial SVM model using the calibration set. In contrast, the training time for the scheme using the N-SVM model included only the time to train the initial SVM model using the training set of Day 1. Furthermore, the response time of a scheme refers to the time it took to classify the EMG feature vectors of the test set of each target day.

The results of the classification accuracy, training time and response time were analyzed using a repeated measure analysis of variance (ANOVA) with factors including classification method (N-SVM, I-SVM, T-SVM, and TI-SVM) and feature selection method (NFS, SFS, and PSO) with a significance level of 0.05. Then, a paired samples *t*-test was performed to compare the classification accuracy of the proposed schemes with that of CNN with fine-tuning. We set the significance level at 0.05. All results were analyzed using IBM^®^ SPSS Statistics 22 software.

## Results

### Dimension Number of the Feature Vectors Selected by Different Feature Selection Methods

The dimensions of the feature vectors of Day 1 selected by different feature selection methods for each subject were reported (Table 1).

**Table 1:** Dimension numbers of the feature vectors selected by different feature selection methods.

	**Dimension number of feature vectors**		
	**NFS**	**SFS**	**PSO**
Subject 1	294	252	36
Subject 2	294	287	59
Subject 3	294	294	102
Subject 4	294	245	70
Subject 5	294	294	78
Subject 6	294	252	77
Subject 7	294	287	92
Subject 8	294	252	58
Subject 9	294	252	113
Subject 10	294	294	62
Subject 11	294	287	138
Subject 12	294	287	90

The results of multiple comparisons showed that the dimension number of the feature vectors selected by the NFS method was significantly higher than those of the SFS (*p* < 0.017) and PSO (*p* < 10^−3^) methods. Similarly, the dimension number of the feature vectors selected by the SFS method was significantly higher than that of the PSO method (*p* < 10^−3^).

### Classification Accuracy of Different EMG Pattern Recognition Schemes

**Figure 4:**
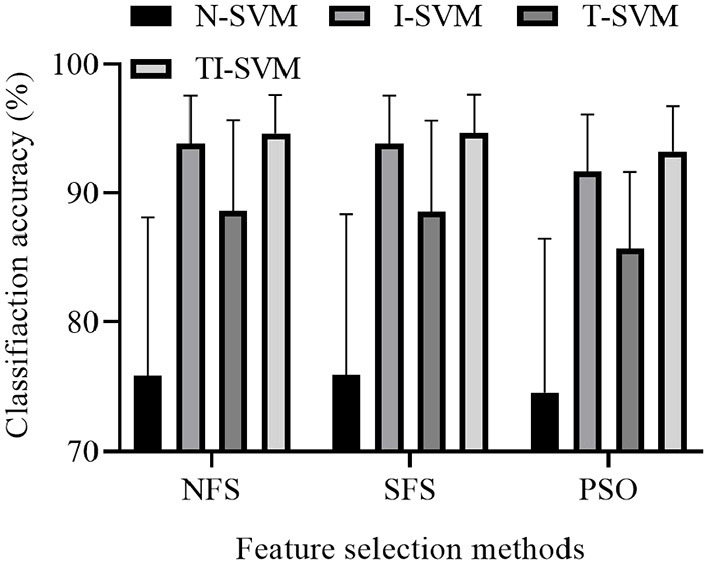
Average classification accuracy of 12 schemes across 12 subjects.

We reported the classification accuracy of 12 schemes that combined three feature selection methods and four classification methods (Figure 4).

The ANOVA analysis indicated that both classification method [*F*_(3,9)_ = 15.692, *p* = 0.001] and feature selection method [*F*_(2,10)_ = 14.134, *p* = 0.001] had a significant effect on the classification accuracy of the 12 combinations. No significant interaction was found between classification method and feature selection method [*F*_(6,6)_ = 3.880, *p* = 0.062].

Multiple comparison results showed that the classification accuracy of TI-SVM was significantly higher than those of N-SVM (*p* = 0.001), I-SVM (*p* = 0.011), and T-SVM (*p* = 0.001). The classification accuracy of T-SVM was significantly higher than that of N-SVM (*p* = 0.015). However, the classification accuracy of T-SVM was significantly lower than that of I-SVM (*p* = 0.001). The classification accuracy of I-SVM was significantly higher than that of N-SVM (*p* = 0.001).

Multiple comparison results showed that the classification accuracy of the scheme trained with the feature vectors selected by the NFS method was significantly higher than that of the same scheme trained with the feature vectors selected by the PSO method (*p* = 0.001). There was no significant difference in classification accuracy between the scheme trained with the feature vectors selected by the NFS method and the scheme trained with the feature vectors selected by the SFS method (*p* = 0.999). However, the classification accuracy of the scheme trained with the feature vectors selected by the SFS method was significantly higher than that of the scheme trained with the feature vectors selected by the PSO method (*p* = 0.001).

### Time Consumption of Different EMG Pattern Recognition Scheme

**Figure 5:**
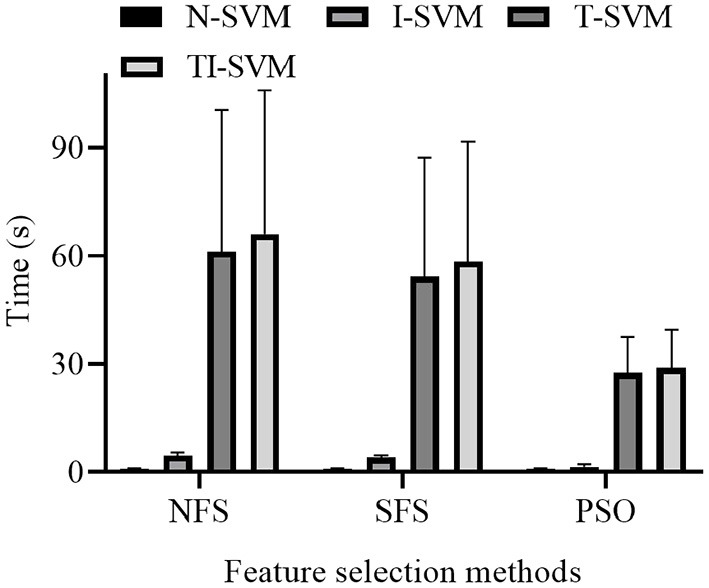
Average training time of different EMG pattern recognition schemes of 12 subjects.

The training times of 12 schemes that combined three feature selection methods and four classification methods were reported (Figure 5).

The ANOVA analysis indicated that both classification method [*F*_(3,9)_ = 198.828, *p* < 10^−3^] and feature selection method [*F*_(2,10)_ = 6.308, *p* = 0.017] had a significant influence on the training time of the schemes. Because a significant interaction between classification method and feature selection method was noted, we further analyzed the simple effect between the classification method and feature selection method [*F*_(6,6)_ = 527.099, *p* < 10^−3^].

The analysis of the simple effect between classification method and feature selection method revealed that classification method had a significant effect on the training time of the schemes trained with the feature vectors selected by NFS [*F*_(3,9)_ = 334.913, *p* < 10^−3^], SFS [*F*_(3,9)_ = 300.485, *p* < 10^−3^], or PSO [*F*_(3,9)_ = 24.636, *p* < 10^−3^] method.

Under three feature selection methods, the training time of TI-SVM was significantly longer than those of N-SVM (*p* < 0.005), I-SVM (*p* < 0.005), and T-SVM (*p* < 10^−3^). The training time of T-SVM was significantly longer than those of N-SVM (*p* < 0.005) and I-SVM (*p* < 0.005). The training time of I-SVM was significantly longer than that of N-SVM (*p* < 10^−3^).

A simple effect analysis revealed that feature selection method had a significant effect on the training time of N-SVM [*F*_(2,10)_ = 472.402, *p* < 10^−3^], I-SVM [*F*_(2,10)_ = 492.173, *p* < 10^−3^], T-SVM [*F*_(2,10)_ = 5.220, *p* = 0.028], and TI-SVM [*F*_(2,10)_ = 6.385, *p* = 0.016].

Under N-SVM, I-SVM, and TI-SVM, the training time of the scheme trained with the feature vectors selected by NFS method was significantly longer than those of the schemes trained with the feature vectors selected by SFS (*p* < 0.05) or PSO (*p* < 0.05) method. The training time of the scheme trained with the feature vectors selected by the SFS method was significantly longer than that of the scheme trained with the feature vectors selected by the PSO method (*p* < 0.05).

Under T-SVM, there was no significant difference in training time between the scheme trained with the feature vectors selected by the NFS method and the scheme trained with the feature vectors selected by the SFS method (*p* = 0.056). The training time of the scheme trained with the feature vectors selected by the NFS method was significantly longer than that of the scheme trained with the feature vectors selected by the PSO method (*p* = 0.018). The training time of the scheme trained with the feature vectors selected by the PSO method was significantly shorter than that of the scheme trained with the feature vectors selected by the SFS method (*p* = 0.02).

**Figure 6:**
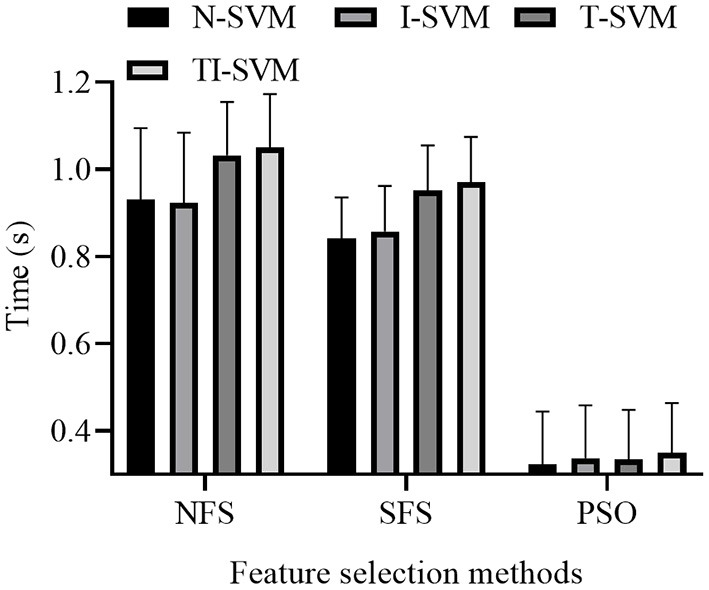
Average response time of different EMG pattern recognition scheme of 12 subjects.

The response time of 12 schemes that combined three feature selection methods and four classification methods were reported (Figure 6).

A repeated measures ANOVA on response time showed a significant effect of classification method [*F*_(3,9)_ = 14.989, *p* = 0.001] and feature selection method [*F*_(2,10)_ = 373.092, *p* < 10^−3^]. There was a significant interaction between classification method and feature selection method [*F*_(6,6)_ = 16.184, *p* = 0.002]. Thus, we further analyzed the simple effect between classification method and feature selection method.

The analysis of the simple effect between classification method and feature selection method revealed that classification method had a significant effect on the response time of the schemes trained with the features selected by the NFS [*F*_(3,9)_ = 14.951, *p* = 0.001], SFS [*F*_(3,9)_ = 8.141, *p* = 0.006], or PSO [*F*_(3,9)_ = 12.535, *p* = 0.001] method.

Under the NFS and SFS methods, the response time of TI-SVM was significantly longer than those of N-SVM (*p* < 0.01), I-SVM (*p* < 0.01), and T-SVM (*p* < 0.01). The response time of T-SVM was longer than those of N-SVM (*p* < 0.02) and I-SVM (*p* < 0.02). There was no significant difference in response time between N-SVM and I-SVM (*p* > 0.5).

Under the PSO method, the response time of TI-SVM was similar to those of N-SVM (*p* = 0.137) and I-SVM (*p* = 0.999). The response time of TI-SVM was significantly longer than that of T-SVM (*p* = 0.001). The response time of T-SVM was similar to those of N-SVM (*p* = 0.999) and I-SVM (*p* = 0.999). The response time of I-SVM was significantly longer than that of N-SVM (*p* = 0.001).

The analysis of the simple effect between classification method and feature selection method revealed that feature selection method had a significant effect on the response time of N-SVM [*F*_(2,10)_ = 286.747, *p* < 10^−3^], I-SVM [*F*_(2,10)_ = 405.599, *p* < 10^−3^], T-SVM [*F*_(2,10)_ = 340.710, *p* < 10^−3^], and TI-SVM [*F*_(2,10)_ = 356.841, *p* < 10^−3^].

Under all classification methods, the response time of the scheme trained with the feature vectors selected by the NFS method were significantly longer than that of the same scheme trained with the feature vectors selected by the PSO method (*p* < 10^−3^). However, there was no significant difference in response time between the scheme trained with the feature vectors selected by the NFS method and the scheme trained with the feature vectors selected by the SFS method (*p* > 0.05). The response time of the scheme trained with the feature vectors selected by the SFS method was significantly longer than that of the same scheme trained with the feature vectors selected by the PSO method (*p* < 10^−3^).

### Comparison of CNN With Fine-Tuning and TI-SVM

As TI-SVM achieved the highest classification accuracy among the four classification methods, to verify the effectiveness of TI-SVM, we compared the classification accuracy of TI-SVM trained with feature vectors selected by different feature selection methods with that of CNN with fine-tuning (Table 2). Analysis of a paired samples *t*-test revealed that the classification accuracy of TI-SVM trained with the features selected by the NFS (*p* = 0.001) or SFS (*p* = 0.001) method was significantly higher than that of CNN with fine-tuning. However, there was no significant difference in classification accuracy between TI-SVM trained with the feature vectors selected by PSO and CNN with fine-tuning (*p* = 0.111).

**Table 2:** Comparison of classification accuracy of CNN with fine-tuning and TI-SVM trained with feature vectors selected by different feature selection methods.

**Classification accuracy (%)**			
**CNN with fine-tuning**	**NFS-TI-SVM**	**SFS-TI-SVM**	**PSO-TI-SVM**
92.00 ± 2.99	94.59 ± 3.00	94.63 ± 2.98	93.23 ± 3.47

## Discussion

In this study, the classification performance of 12 combinations of three feature selection methods and four classification methods on 4 target days were compared to find a proper EMG pattern recognition scheme for repeated uses. Our findings revealed that the TI-SVM trained with the feature vectors selected by SFS achieved a considerable performance in classification accuracy and reduction in training time. TI-SVM achieved the highest classification accuracy among the four classification methods. Moreover, TI-SVM can improve the performance of I-SVM by discarding outdated data from the training set and focusing on data from the calibration set, which are difficult to classify correctly. Compared with the NFS method, the SFS method is a robust feature selection method that can reduce the training time of TI-SVM and maintain the classification accuracy of the scheme in repeated uses. Although the PSO method outperformed the SFS method in the reduction in training and response times, it significantly reduced the classification accuracy of the scheme trained with the feature vectors selected by the NFS or SFS methods. Thus, TI-SVM combined with the SFS method is a proper scheme for EMG pattern recognition in repeated uses. Most importantly, TI-SVM trained with the feature vectors selected by the SFS method required only a little amount of data (only 5 s of data per motion was used to adapt the model) to adapt the model and achieved considerable classification accuracy that outperformed CNN with fine-tuning.

TI-SVM is a suitable classification method for the condition of electrodes shifting in repeated uses when compared with the other three tested classification methods. I-SVM maintained a considerable classification accuracy when compared with N-SVM over 4 target days. This finding is consistent with that of a previous study (Liang and Li, 2009). Thus, I-SVM was a robust classification method for EMG pattern recognition despite the electrodes shifting across every use. However, the classification accuracy of I-SVM was significantly lower when compared with that of TI-SVM. A previous study indicated that outdated samples in the training set would decrease the performance of I-SVM (Huang et al., 2017). TrAdaBoost can solve this problem by discarding the samples whose distribution in the training set is different from that in the test set, and selecting the samples that are difficult to classify correctly in the test set (Matasci et al., 2012). Although the classification accuracy of T-SVM was lower than that of I-SVM, the TrAdaBoost algorithm indeed played a role in improving the classification accuracy of I-SVM in repeated uses. Previous studies using transfer learning to solve the decline in classification accuracy due to data with different distributions have achieved many considerable achievements (Liu et al., 2016; Vidovic et al., 2016; Ameri et al., 2020). Our research combined transfer learning and incremental learning methods and proved that transfer learning can enhance the performance of incremental learning. Thus, we speculate that transfer learning can also play a role in improving the classification accuracy of the other supervised adaptive classification methods such as CNN with fine-tuning. Moreover, previous studies only adopted adaptive classification methods to solve the problem of classification accuracy decline due to electrodes shifting and ignored the application to the real-time performance of the scheme. The real-time performance of the adaptive classification methods should be considered in more depth. With the increase in new data and the iterations of adaptive classification methods, the model becomes more complex. The complex model increases the time consumption of the scheme. Therefore, the time consumption of the adaptive classification method needs to be further investigated.

In this study, two widely used feature selection methods were used to reduce the dimensions of EMG feature vectors to improve the real-time performance of the classification schemes. The NFS method served as the base line method to evaluate the effectiveness of the two methods. Our findings indicate that the schemes trained with the feature vectors selected by the SFS or PSO methods did not improve the classification accuracy compared with that of the feature vectors selected by the NFS method in repeated uses. These findings are inconsistent with a previous study that stated that feature selection can improve the classification accuracy of an EMG pattern recognition scheme (Liu, 2014; Adewuyi et al., 2016; Purushothaman and Vikas, 2018). Unlike the previous studies, our study includes the factor of electrodes shifting in repeated uses. We think it more appropriate to discuss the robustness of the feature vectors selected by different feature selection methods for clinical application. The robustness of the feature vectors selected by the PSO method was weaker than that of the feature vectors selected by the SFS method in repeated uses. This finding may be due to the fact that the PSO method selected fewer informative dimensions of EMG feature vectors than did the SFS method, which increased the difference in data distribution between different uses. The SFS method can select more information from the original high-dimensional feature vectors and maintain the robustness of EMG feature vectors across repeated uses. For time consumption reduction, the schemes trained with the feature vectors selected by the PSO method consumed a significantly shorter training time and response time than those of the schemes trained with the feature vectors selected by the NFS or SFS methods. Although the PSO method outperformed the NFS and SFS methods in reducing the training and response times, the PSO method considerably reduced the classification accuracy of the schemes trained with the feature vectors selected by the NFS or SFS method. The SFS method can maintain the robustness of the original feature vectors while reducing the training time of TI-SVM. Thus, the SFS method is a suitable feature selection method for TI-SVM in repeated uses.

Previous studies adopted deep learning technologies for EMG pattern recognition (Ameri et al., 2020). However, it is difficult to collect a huge amount of data from a single subject to train a considerably deep network. If deep learning techniques are applied to the classification of a small sample data set, overfitting will occur. Establishing a large data set with data from different subjects or using a few-shot learning method can address this problem; however, this decreases the performance of the classification model and increases the difficulty of training the model. In this study, we trained TI-SVM with SFS on a small data set for each subject and achieved a considerable performance that outperformed CNN with fine-tuning.

## Conclusion

In this study, we optimized the feature selection and classification methods in the EMG pattern recognition scheme to develop a suitable scheme that combines the TI-SVM model with the SFS method to improve the robustness of the scheme in repeated uses. The developed scheme not only maintained its classification accuracy in different uses, but also provided a considerable real-time performance. The method also demonstrated good performance on a small EMG data set, which implies that a user need not collect voluminous EMG data to train a classification model. Based on the findings of our study, TrAdaBoost significantly improves the performance of I-SVM. Thus, we speculate that transfer learning can also improve the performance of supervised adaptive classification methods using deep learning methods. Nevertheless, in this study, we ignored the influence of long-term use without electrode shifting and different muscle contraction levels on the EMG pattern recognition scheme. Thus, in a future work, the effectiveness of the proposed scheme will be verified under the influence of combined factors.

## Data Availability Statement

The raw data supporting the conclusions of this article will be made available by the authors, without undue reservation.

## Ethics Statement

The studies involving human participants were reviewed and approved by Ethics committee of Changchun University of Science and Technology (CUST). The patients/participants provided their written informed consent to participate in this study.

## Author Contributions

QL designed experiments. AZ performed experiments. AZ and ZL analyzed experimental results. YW check and verified the experimental results. QL and AZ wrote the manuscript. All authors have contributed to wrote this manuscript and approved the final version of the manuscript.

## Conflict of Interest

The authors declare that the research was conducted in the absence of any commercial or financial relationships that could be construed as a potential conflict of interest.
